# Twins Locked in Each Other

**Published:** 1849

**Authors:** 


### ARTICLE II.

*Twins locked in each other.* By Prof. MURPHY.—(*Med. Gaz.*)

A complication of a singular character has been recorded in which delivery was rendered extremely difficult. The late Dr. T. Ferguson, of Dublin, relates a case of twins in which the first child presented the foot, and was delivered without any unusual obstacle in the progress of the labor until the child's body was so far protruded as to enable Dr. Ferguson to ascertain, by the pulsation of the funis, then without the os externum, that the child was alive. From this stage of the delivery he began to experience a most unusual and unaccountable resistance to the further descent of the child. This difficulty was produced by the head of the second child descending before the head of the first, so that each locked in the other. The pulsation in the funis of the first child still continuing, Dr. Ferguson wished to perforate the head of the second, that caused the obstruction: there was some delay in obtaining instruments; and in the interval, the pulsation of the first child ceased; but, to the surprise of Dr. Ferguson, powerful expulsive pains forced down the heads of both over the perineum, and the second child was born living! Two years ago Mr. Elton, of Windsor, related a similar case. The feet of the first child presented, and were brought down, but, "after the thighs had passed, the delivery became slow and increasingly difficult; the abdomen suffered great compression in passing; the thorax still more; the difficulty became greater with the further progress of the body; the arms were extracted with much trouble, and, it then being practicable, an examination was made. I found the vertex of a full-sized head presenting immediately over the breast in the position where there should have been a chin; the anterior base of the neck conld be traced in close and compressed contact with the presenting head, the latter firmly impacted in the pelvic cavity." Mr. Eton divided the neck of the first child; and, having removed the truncated body, applied the forceps to the second child, which he delivered, but could not save, although attempts "to restore animation were long and anxiously continued." What is to be done in such a case as this? I certainly should not be disposed to destroy either child. Before I took up the peforator or the amputating knife, I should weigh well the practibility of applying the long forceps to the head of the second child, and endeavor to imitate nature in the effort to force both heads over the perineum. If you succeeded, its laceration might be the consequence; but it would be some recompense to save a life that otherwise you must destroy."—*N. Y. Jour. of Med.*

